# The genome sequence of a riffle beetle, *Elmis aenea *(Müller, 1806)

**DOI:** 10.12688/wellcomeopenres.19778.1

**Published:** 2023-07-26

**Authors:** John D.S. Findlay, Garth Foster, Natural History Museum Genome Acquisition Lab, Darwin Tree of Life Barcoding collective, Wellcome Sanger Institute Tree of Life programme, Wellcome Sanger Institute Scientific Operations: DNA Pipelines collective, Tree of Life Core Informatics collective, Darwin Tree of Life Consortium

**Affiliations:** 1 Environment Agency, Huntingdon, England, UK; 2 Aquatic Coleoptera Conservation Trust, Ayr, Scotland, UK

**Keywords:** Elmis aenea, riffle beetle, genome sequence, chromosomal, Coleoptera

## Abstract

We present a genome assembly from an individual female *Elmis aenea* (a riffle beetle; Arthropoda; Insecta; Coleoptera; Elmidae). The genome sequence is 516.5 megabases in span. Most of the assembly is scaffolded into 9 chromosomal pseudomolecules, including the X sex chromosome. The mitochondrial genome has also been assembled and is 18.06 kilobases in length.

## Species taxonomy

Eukaryota; Metazoa; Eumetazoa; Bilateria; Protostomia; Ecdysozoa; Panarthropoda; Arthropoda; Mandibulata; Pancrustacea; Hexapoda; Insecta; Dicondylia; Pterygota; Neoptera; Endopterygota; Coleoptera; Polyphaga; Elateriformia; Byrrhoidea; Elmidae; Elminae; *Elmis*; *Elmis aenea* (Müller, 1806) (NCBI:txid186982).

## Background

**Figure 1.**
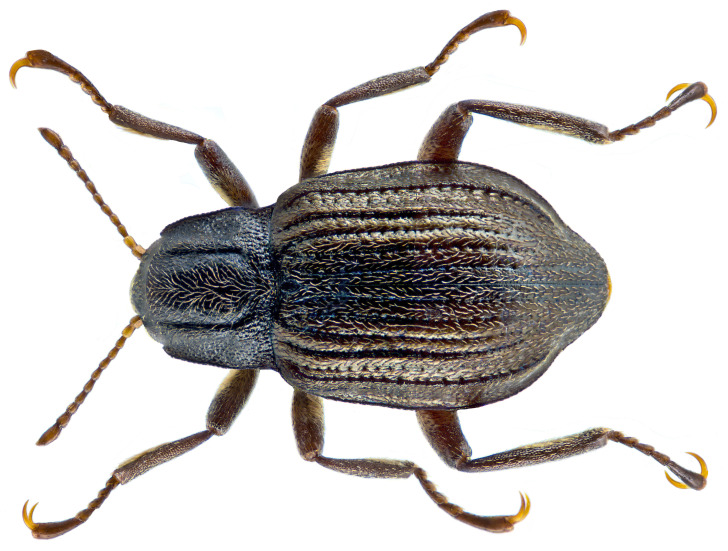
Photograph of *Elmis aenea* (icElmAene2). Photograph by Udo Schmidt (CC BY-SA 2.0).

*Elmis aenea* ( Figure 1) is a western Palaearctic species found from Portugal to Finland, Russia and Ukraine ( Jäch *et al.*, 2016). It is the commonest elmid beetle in Britain and Ireland, known in most parts except the Shetlands, Uists, Coll and Tiree, and the Isle of Man ( Foster *et al.*, 2020). It is, however, rare in some lowland areas lacking fast-running water, such as the Wash, and in heavily industrialised or lowland urbanised areas such as around Manchester, the Tyne Valley and parts of West Yorkshire ( Hammond, 2017). Whereas it might be considered almost eurythermal in Britain and Ireland, found in any fast-running water, in mainland Europe it reaches 2,400 metres above sea level in Italy ( Olmi, 1976), and is generally considered to be an upland species, but this view has been challenged in Poland ( Buczyński & Buczyńska, 2022). It is, however, most likely to be found in headwaters, often in the absence of other riffle beetle species, and it is occasionally found in subterranean waters ( Knight, 2011). Adults were found in areas with a particle size averaging 103 mm in diameter whereas larvae were found where particle size averaged 77 mm ( Malmqvist & Sjöström, 1984). Elliott (2008) found that about half of adults tested would fly, but flight records are rare in nature, and this tallies with the beetle's absence from the Isle of Man, where the other three common elmid species occur, as originally reported by ( Britten, 1944).

Adults and larvae are detritivorous but adult guts may also contain periphytic algae ( Elliott, 2008). Eggs are laid from April to June on aquatic mosses, there being six instars with mostly the fourth one overwintering ( Holland, 1972). The final instar leaves the water to pupate in the following spring, with a new generation of adults emerging that autumn. Adults and larvae can be encountered together through most of the year, except when adults are scarce in January and February. Decline has been reported in association with milder winter temperatures and higher than average rainfall during the cycle of the North Atlantic Oscillation ( Bradley & Ormerod, 2001). Life-table analysis by ( Elliott, 2006) showed that the greatest losses, at 36%, are in immature stages owing to spating. On the other hand, ( Hoffsten, 2003) noted slow recovery following a severe winter in Sweden when precipitation was low.

*Elmis aenea* is the sole known representative of *Elmis* in Britain and Ireland. Several other *Elmis* species, in particular *E. maugetii* Latreille, can be found in low-lying parts of mainland Europe ( Olmi, 1976). There is a faint possibility that their presence has been overlooked if anywhere in eastern England. The high-quality genome sequence described here is, to our knowledge, the first one reported for the true *E. aenea*. The genome sequence for *E. aenea* will aid in understanding the biology, physiology and ecology of the species.

## Genome sequence report

The genome was sequenced from one female *Elmis aenea* collected from Great Staughton, UK (52.27, –0.35). A total of 35-fold coverage in Pacific Biosciences single-molecule HiFi long reads was generated. Primary assembly contigs were scaffolded with chromosome conformation Hi-C data. Manual assembly curation corrected 89 missing joins or misjoins, reducing the scaffold number by 4.57%, and increasing the scaffold N50 by 0.34%.

**Figure 2.**
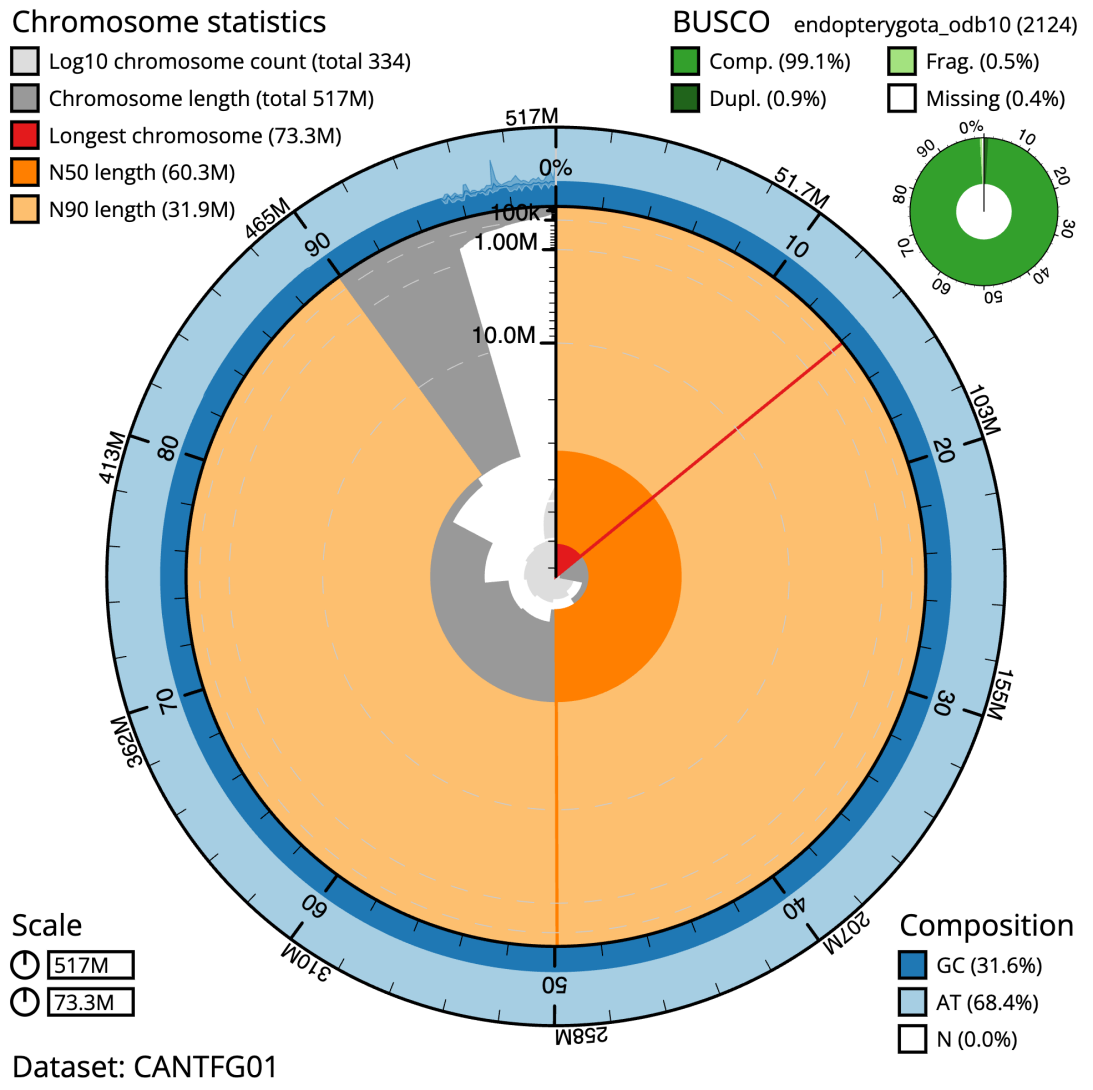
Genome assembly of *Elmis aenea*, icElmAene2.1: metrics. The BlobToolKit Snailplot shows N50 metrics and BUSCO gene completeness. The main plot is divided into 1,000 size-ordered bins around the circumference with each bin representing 0.1% of the 516,518,598 bp assembly. The distribution of scaffold lengths is shown in dark grey with the plot radius scaled to the longest scaffold present in the assembly (73,284,568 bp, shown in red). Orange and pale-orange arcs show the N50 and N90 scaffold lengths (60,347,194 and 31,928,154 bp), respectively. The pale grey spiral shows the cumulative scaffold count on a log scale with white scale lines showing successive orders of magnitude. The blue and pale-blue area around the outside of the plot shows the distribution of GC, AT and N percentages in the same bins as the inner plot. A summary of complete, fragmented, duplicated and missing BUSCO genes in the endopterygota_odb10 set is shown in the top right. An interactive version of this figure is available at https://blobtoolkit.genomehubs.org/view/Elmis aenea/dataset/CANTFG01/snail.

The final assembly has a total length of 516.5 Mb in 333 sequence scaffolds with a scaffold N50 of 60.3 Mb ( Table 1). Most (95.3%) of the assembly sequence was assigned to 9 chromosomal-level scaffolds, representing 8 autosomes and the X sex chromosome. Chromosome-scale scaffolds confirmed by the Hi-C data are named in order of size ( Figure 2– Figure 5; Table 2). The X chromosome was identified based on synteny with *Agrypnus murinus* (GCA_929113105.1; icAgrMuri1.1). While not fully phased, the assembly deposited is of one haplotype. Contigs corresponding to the second haplotype have also been deposited. The mitochondrial genome was also assembled and can be found as a contig within the multifasta file of the genome submission.

**Table 1.** Genome data for *Elmis aenea*, icElmAene2.1.

Project accession data		
Assembly identifier	icElmAene2.1	
Species	*Elmis aenea*	
Specimen	icElmAene2	
NCBI taxonomy ID	186982	
BioProject	PRJEB57664	
BioSample ID	SAMEA7521014	
Isolate information	icElmAene2, female: whole organism (DNA sequencing); icElmAene3, whole organism (Hi-C scaffolding)	
Assembly metrics *		*Benchmark*
Consensus quality (QV)	58.1	*≥ 50*
*k*-mer completeness	99.99%	*≥ 95%*
BUSCO **	C:99.1%[S:98.2%,D:0.9%], F:0.5%,M:0.4%,n:2,124	*C ≥ 95%*
Percentage of assembly mapped to chromosomes	95.3%	*≥ 95%*
Sex chromosomes	X chromosome	*localised homologous pairs*
Organelles	Mitochondrial genome assembled.	*complete single alleles*
Raw data accessions		
PacificBiosciences SEQUEL II	ERR10499354	
Hi-C Illumina	ERR10501012	
Genome assembly		
Assembly accession	GCA_947652605.1	
*Accession of alternate haplotype*	GCA_947652985.1	
Span (Mb)	516.5	
Number of contigs	1187	
Contig N50 length (Mb)	0.9	
Number of scaffolds	333	
Scaffold N50 length (Mb)	60.3	
Longest scaffold (Mb)	73.3	

* Assembly metric benchmarks are adapted from column VGP-2020 of “Table 1: Proposed standards and metrics for defining genome assembly quality” from ( Rhie *et al.*, 2021). ** BUSCO scores based on the endopterygota_odb10 BUSCO set using v5.3.2. C = complete [S = single copy, D = duplicated], F = fragmented, M = missing, n = number of orthologues in comparison. A full set of BUSCO scores is available at https://blobtoolkit.genomehubs.org/view/Elmis%20aenea/dataset/CANTFG01/busco.

**Figure 3.**
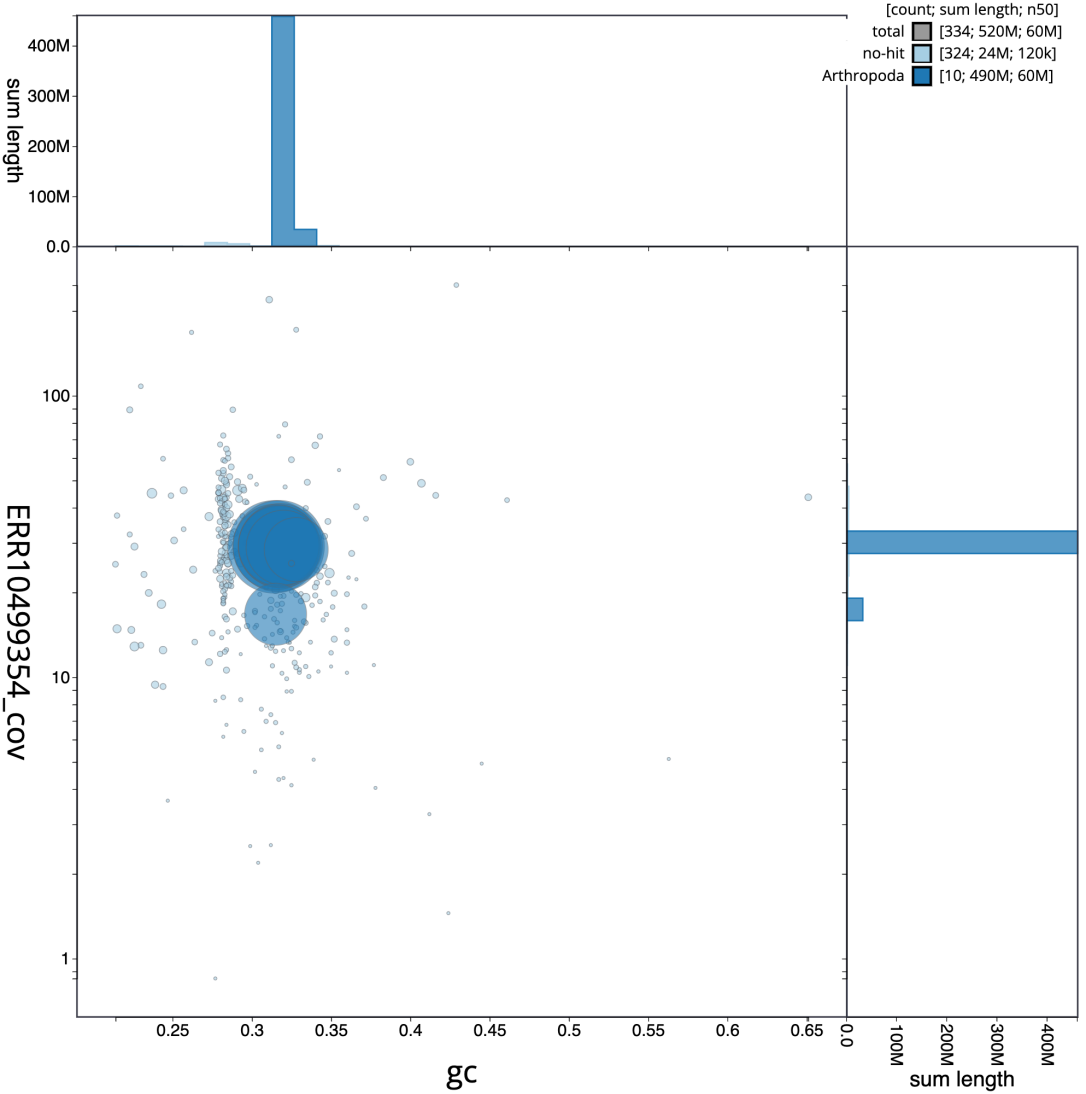
Genome assembly of *Elmis aenea*, icElmAene2.1: BlobToolKit GC-coverage plot. Scaffolds are coloured by phylum. Circles are sized in proportion to scaffold length. Histograms show the distribution of scaffold length sum along each axis. An interactive version of this figure is available at https://blobtoolkit.genomehubs.org/view/Elmis%20aenea/dataset/CANTFG01/blob.

**Figure 4.**
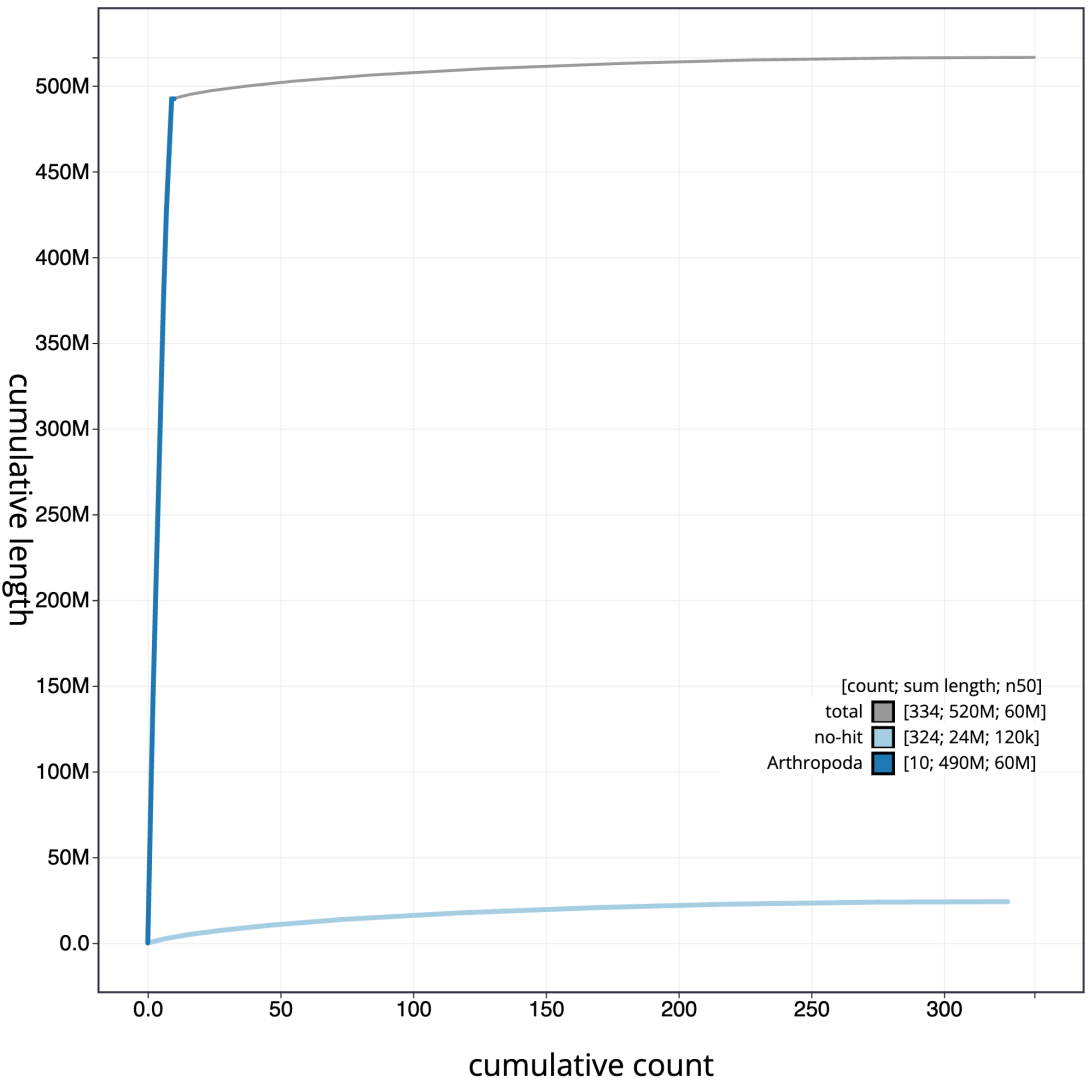
Genome assembly of *Elmis aenea*, icElmAene2.1: BlobToolKit cumulative sequence plot. The grey line shows cumulative length for all scaffolds. Coloured lines show cumulative lengths of scaffolds assigned to each phylum using the buscogenes taxrule. An interactive version of this figure is available at https://blobtoolkit.genomehubs.org/view/Elmis aenea/dataset/CANTFG01/cumulative.

**Figure 5.**
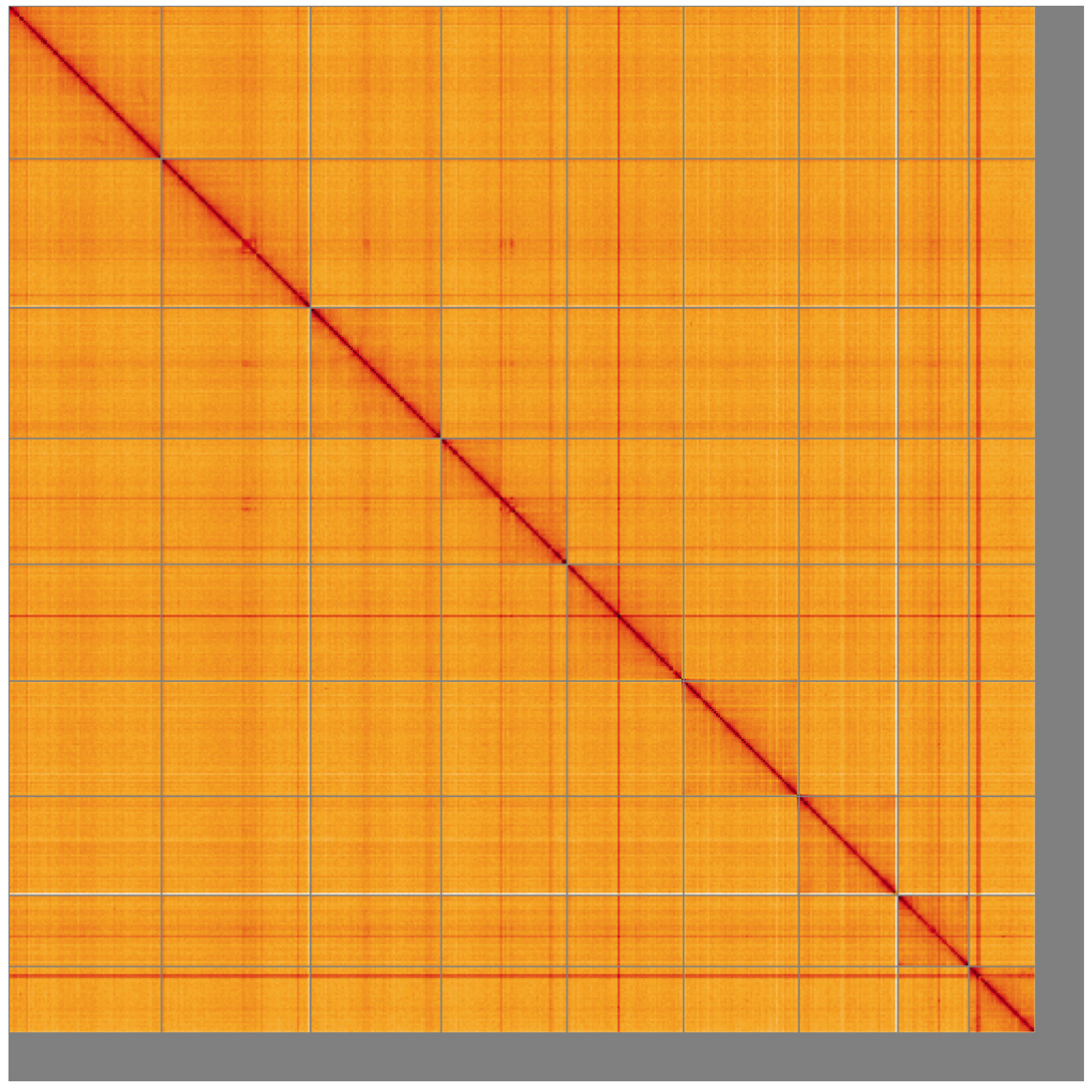
Genome assembly of *Elmis aenea*, icElmAene2.1: Hi-C contact map of the icElmAene2.1 assembly, visualised using HiGlass. Chromosomes are shown in order of size from left to right and top to bottom. An interactive version of this figure may be viewed at https://genome-note-higlass.tol.sanger.ac.uk/l/?d=FN93Vc1kQT6dWcM8J2qaVw.

**Table 2.** Chromosomal pseudomolecules in the genome assembly of *Elmis aenea*, icElmAene 2.

INSDC accession	Chromosome	Length (Mb)	GC%
OX393576.1	1	73.28	31.5
OX393577.1	2	71.35	31.5
OX393578.1	3	62.65	31.5
OX393579.1	4	60.35	32.0
OX393580.1	5	55.9	31.5
OX393581.1	6	55.28	31.5
OX393582.1	7	47.42	32.0
OX393583.1	8	34.12	33.0
OX393584.1	X	31.93	31.5
OX393585.1	MT	0.02	27.0

The estimated Quality Value (QV) of the final assembly is 58.1 with *k*-mer completeness of 99.99%, and the assembly has a BUSCO v5.3.2 completeness of 99.1% (single = 98.2%, duplicated = 0.9%), using the endopterygota_odb10 reference set ( *n* = 2,124).

Metadata for specimens, spectral estimates, sequencing runs, contaminants and pre-curation assembly statistics can be found at https://links.tol.sanger.ac.uk/species/186982.

## Methods

### Sample acquisition and nucleic acid extraction

The specimen used for genome sequencing was a female *Elmis aenea* (specimen ID NHMUK014361123, individual icElmAene2), collected from freshwater in Great Staughton, UK (latitude 52.27, longitude –0.35) on 2019-03-11 using a kick-net. The specimen used for Hi-C scaffolding was icElmAene3, which was collected from freshwater in Dover Beck, Caythorpe, UK (latitude 53.01, longitude –0.99) on 2019-03-19. The specimens were collected and identified by John Findlay (Environment Agency), and were snap-frozen on dry ice.

DNA was extracted at the Tree of Life laboratory, Wellcome Sanger Institute (WSI). The icElmAene2 sample was weighed and dissected on dry ice. Tissue from the whole organism was cryogenically disrupted to a fine powder using a Covaris cryoPREP Automated Dry Pulveriser, receiving multiple impacts. High molecular weight (HMW) DNA was extracted using the Qiagen MagAttract HMW DNA extraction kit. HMW DNA was sheared into an average fragment size of 12–20 kb in a Megaruptor 3 system with speed setting 30. Sheared DNA was purified by solid-phase reversible immobilisation using AMPure PB beads with a 1.8X ratio of beads to sample to remove the shorter fragments and concentrate the DNA sample. The concentration of the sheared and purified DNA was assessed using a Nanodrop spectrophotometer and Qubit Fluorometer and Qubit dsDNA High Sensitivity Assay kit. Fragment size distribution was evaluated by running the sample on the FemtoPulse system.

### Sequencing

Pacific Biosciences HiFi circular consensus DNA sequencing libraries were constructed according to the manufacturers’ instructions. DNA sequencing was performed by the Scientific Operations core at the WSI on a Pacific Biosciences SEQUEL II (HiFi) instrument. Hi-C data were also generated from tissue of icElmAene3 using the Arima2 kit and sequenced on the Illumina NovaSeq 6000 instrument.

### Genome assembly, curation and evaluation

Assembly was carried out with Hifiasm ( Cheng *et al.*, 2021) and haplotypic duplication was identified and removed with purge_dups ( Guan *et al.*, 2020). One round of polishing was performed by aligning 10X Genomics read data to the assembly with Long Ranger ALIGN, calling variants with FreeBayes ( Garrison & Marth, 2012). The assembly was then scaffolded with Hi-C data ( Rao *et al.*, 2014) using YaHS ( Zhou *et al.*, 2023). The assembly was checked for contamination and corrected using the gEVAL system ( Chow *et al.*, 2016) as described previously ( Howe *et al.*, 2021). Manual curation was performed using gEVAL, HiGlass ( Kerpedjiev *et al.*, 2018) and Pretext ( Harry, 2022). The mitochondrial genome was assembled using MitoHiFi ( Uliano-Silva *et al.*, 2023), which runs MitoFinder ( Allio *et al.*, 2020) or MITOS ( Bernt *et al.*, 2013) and uses these annotations to select the final mitochondrial contig and to ensure the general quality of the sequence.

A Hi-C map for the final assembly was produced using bwa-mem2 ( Vasimuddin *et al.*, 2019) in the Cooler file format ( Abdennur & Mirny, 2020). To assess the assembly metrics, the *k*-mer completeness and QV consensus quality values were calculated in Merqury ( Rhie *et al.*, 2020). This work was done using Nextflow ( Di Tommaso *et al.*, 2017) DSL2 pipelines “sanger-tol/readmapping” ( Surana *et al.*, 2023a) and “sanger-tol/genomenote” ( Surana *et al.*, 2023b). The genome was analysed within the BlobToolKit environment ( Challis *et al.*, 2020) and BUSCO scores ( Manni *et al.*, 2021; Simão *et al.*, 2015) were calculated.

Table 3 contains a list of relevant software tool versions and sources.

**Table 3.** Software tools: versions and sources.

Software tool	Version	Source
BlobToolKit	4.1.5	https://github.com/blobtoolkit/blobtoolkit
BUSCO	5.3.2	https://gitlab.com/ezlab/busco
gEVAL	N/A	https://geval.org.uk/
Hifiasm	0.16.1-r375	https://github.com/chhylp123/hifiasm
HiGlass	1.11.6	https://github.com/higlass/higlass
Merqury	MerquryFK	https://github.com/thegenemyers/MERQURY.FK
MitoHiFi	2	https://github.com/marcelauliano/MitoHiFi
PretextView	0.2	https://github.com/wtsi-hpag/PretextView
purge_dups	1.2.3	https://github.com/dfguan/purge_dups
sanger-tol/genomenote	v1.0	https://github.com/sanger-tol/genomenote
sanger-tol/readmapping	1.1.0	https://github.com/sanger-tol/readmapping/tree/1.1.0
YaHS	1.1a.2	https://github.com/c-zhou/yahs

### Wellcome Sanger Institute – Legal and Governance

The materials that have contributed to this genome note have been supplied by a Darwin Tree of Life Partner. The submission of materials by a Darwin Tree of Life Partner is subject to the **‘Darwin Tree of Life Project Sampling Code of Practice’**, which can be found in full on the Darwin Tree of Life website here. By agreeing with and signing up to the Sampling Code of Practice, the Darwin Tree of Life Partner agrees they will meet the legal and ethical requirements and standards set out within this document in respect of all samples acquired for, and supplied to, the Darwin Tree of Life Project.

Further, the Wellcome Sanger Institute employs a process whereby due diligence is carried out proportionate to the nature of the materials themselves, and the circumstances under which they have been/are to be collected and provided for use. The purpose of this is to address and mitigate any potential legal and/or ethical implications of receipt and use of the materials as part of the research project, and to ensure that in doing so we align with best practice wherever possible. The overarching areas of consideration are:

•   Ethical review of provenance and sourcing of the material

•   Legality of collection, transfer and use (national and international) 

Each transfer of samples is further undertaken according to a Research Collaboration Agreement or Material Transfer Agreement entered into by the Darwin Tree of Life Partner, Genome Research Limited (operating as the Wellcome Sanger Institute), and in some circumstances other Darwin Tree of Life collaborators.

## Data Availability

European Nucleotide Archive: *Elmis aenea* (riffle beetle). Accession number PRJEB57664; https://identifiers.org/ena.embl/PRJEB57664. ( Wellcome Sanger Institute, 2022) The genome sequence is released openly for reuse. The *Elmis aenea* genome sequencing initiative is part of the Darwin Tree of Life (DToL) project. All raw sequence data and the assembly have been deposited in INSDC databases. The genome will be annotated using available RNA-Seq data and presented through the Ensembl pipeline at the European Bioinformatics Institute. Raw data and assembly accession identifiers are reported in Table 1.

## Author information

Members of the Natural History Museum Genome Acquisition Lab are listed here: https://doi.org/10.5281/zenodo.4790042.

Members of the Darwin Tree of Life Barcoding collective are listed here: https://doi.org/10.5281/zenodo.4893703.

Members of the Wellcome Sanger Institute Tree of Life programme are listed here: https://doi.org/10.5281/zenodo.4783585.

Members of Wellcome Sanger Institute Scientific Operations: DNA Pipelines collective are listed here: https://doi.org/10.5281/zenodo.4790455.

Members of the Tree of Life Core Informatics collective are listed here: https://doi.org/10.5281/zenodo.5013541.

Members of the Darwin Tree of Life Consortium are listed here: https://doi.org/10.5281/zenodo.4783558.
