# Supplementary material for: The genome sequence of a riffle beetle, Elmis aenea (Müller, 1806)
Source: Wellcome Open Res. 2023 Jul 26;8:322. [Version 1] doi: 10.12688/wellcomeopenres.19778.1 (PMC10660300; doi:10.12688/wellcomeopenres.19778.1)
Supplement: Peer review history

# Reviewer report: Reviewer response for version 1

**DOI:** 10.21956/wellcomeopenres.21906.r70170

**Published:** 2023-11-20

**Authors:** Jerome H.L Hui

**Affiliations:** 1 Chinese University of Hong Kong, Hong Kong, China

Findlay, Foster, and colleagues report the genome sequence of riffle bettle *Elmis aenea *(Müller, 1806). This species can be commonly found in running water throughout mainland UK and Ireland. Molecular data of this species are scarce prior to this report, and are mainly confined to COI sequences deposited to the NCBI database. This new genome resource will be very useful for further studies, such as understanding their ecology, population structure, response to environment/climate changes, and evolutionary relationships with other beetles.

 This genome resource is excellent from the summary statistics, with high BUSCO numbers, high sequence continuity (scaffold N50), and majority of sequences contained on the 9 pseudochromosomes (plus mitochondrion). To sum up, this is a valuable contribution.

Are sufficient details of methods and materials provided to allow replication by others?

Yes

Is the rationale for creating the dataset(s) clearly described?

Yes

Are the datasets clearly presented in a useable and accessible format?

Yes

Are the protocols appropriate and is the work technically sound?

Yes

Reviewer Expertise:

Genomics, evolution, invertebrates

I confirm that I have read this submission and believe that I have an appropriate level of expertise to confirm that it is of an acceptable scientific standard.

# Reviewer report: Reviewer response for version 1

**DOI:** 10.21956/wellcomeopenres.21906.r64770

**Published:** 2023-08-08

**Authors:** Susan Brown

**Affiliations:** 1 KSU Bioinformatics Center, Division of Biology, Kansas State University, Manhattan, Kansas, USA

This data note on the genome sequence of the riffle beetle follows standard protocols for specimen collection, DNA extraction, sequencing, assembly and QC. The background includes several unrelated facts, such as the average particle size in the environment of adults and larvae. Although climate change has increased spating globally, your readers on the other side of the pond might appreciate not having to look it up. There are two references at the end of the second paragraph where the authors names should be outside the parentheses.

Are sufficient details of methods and materials provided to allow replication by others?

Yes

Is the rationale for creating the dataset(s) clearly described?

Yes

Are the datasets clearly presented in a useable and accessible format?

Yes

Are the protocols appropriate and is the work technically sound?

Yes

Reviewer Expertise:

Insect developmental genetics

I confirm that I have read this submission and believe that I have an appropriate level of expertise to confirm that it is of an acceptable scientific standard.
